# Acute myocardial infarction in adolescents: reappraisal of underlying mechanisms

**DOI:** 10.1007/s12471-020-01408-y

**Published:** 2020-03-18

**Authors:** G. G. F. van der Schoot, R. L. Anthonio, G. A. J. Jessurun

**Affiliations:** Department of Cardiology, Treant Zorggroep Scheper Ziekenhuis, Emmen, The Netherlands

**Keywords:** ST-elevation myocardial infarction, Acute coronary syndrome, Primary percutaneous coronary intervention, Risk factors

## Abstract

Worldwide, a myocardial infarction (MI) is an important cause of death. Acute MI occurs most commonly at an older age. However, the incidence of acute MI in adolescents is increasing. This is partly due to an increase in cardiovascular risk factors (e.g. smoking, unhealthy diet), which might lead to premature atherosclerosis. However, several non-atherosclerotic causes of MI in adolescents are also described in the literature, such as vascular spasm due to the use of cocaine. We may assume that acute MI is not considered to be the most likely cause of chest pain in adolescents. Therefore, the risk of a dramatic outcome in this patient category may be significant. This point of view article addresses the pathophysiological process and subsequent diagnostic approach in adolescents with MI resulting from either premature atherosclerosis or of non-atherosclerotic causes. Insight into the potential operational mechanisms of the coronary artery incident may have a major impact on the clinical course following admission. We would like to underline that a personalised clinical approach remains of utmost importance in each patient treated by protocolised medicine. This is particularly true when acute MI occurs at a young age, since the underlying cause more frequently differs from the conventional atherosclerotic process in this patient category.

## Introduction

Worldwide, a myocardial infarction (MI) is an important cause of death. Fortunately, acute MI in young patients is rare (<10% of patients are younger than 40 years) [1]. However, the prevalence of acute MI in young patients is increasing. We may assume that acute MI is not considered to be the most likely cause of chest pain in adolescents. Therefore, the risk of a dramatic outcome in this patient category may be significant.

Acute MI in adolescents may be caused by premature atherosclerosis or have non-atherosclerotic causes (e.g. vascular spasms during the use of cocaine) [2]. This point of view article addresses the pathophysiological process and the diagnostic approach in adolescents with MI resulting from either premature atherosclerosis or non-atherosclerotic causes. We would like to mention that our description of non-atherosclerotic causes extends beyond the current clinical conception of MINOCA (myocardial infarction in non-obstructive coronary artery disease) [3]. First, we describe two cases of adolescents who developed acute MI.

## Case reports

### Case 1

**Fig. 1:**
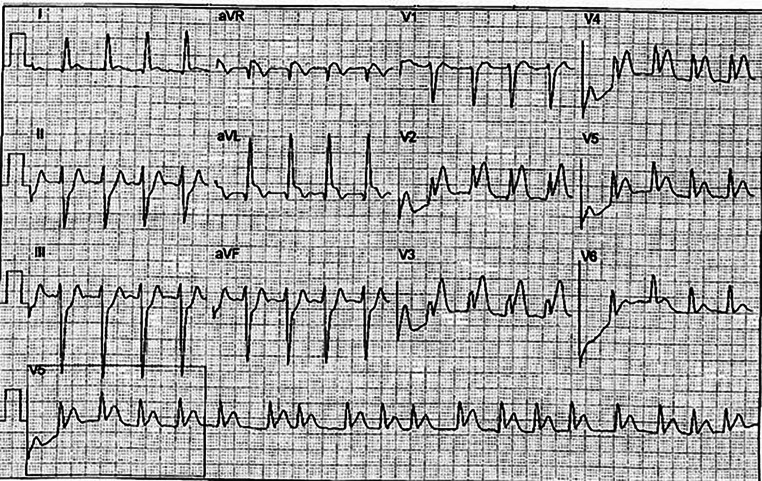
Acute transmural ischaemia in the anterior wall with an expected occlusion before branching of the left anterior descending coronary artery

**Fig. 2:**
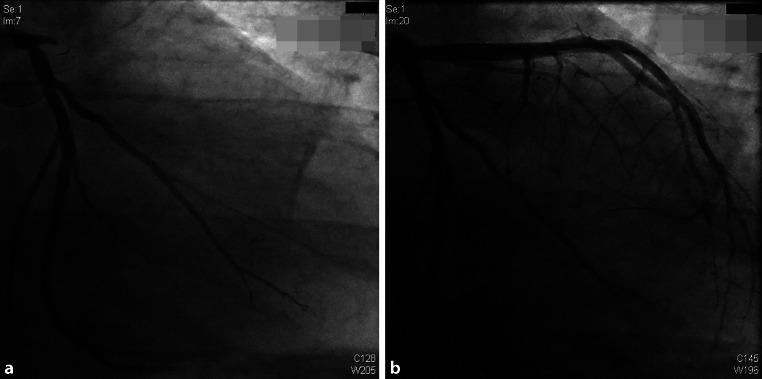
**a** Left anterior descending coronary artery, occlusion at the ostium. **b** Result immediately after percutaneous coronary intervention

A 21-year-old male was admitted to the hospital because of pain in the middle of his chest, radiating to his left arm and between the scapulae. He felt a tingling sensation in his arm, fingers and lips and had a sore feeling in his jaw. The patient perspired heavily and he was very short of breath. In the previous 2 months, the sore feeling in his jaw and arm had also occurred intermittently. He had no medical history. In the previous 2 years, the patient had smoked 15 cigarettes a day. Cardiovascular disease at a younger age was present in his family. His blood pressure was 128/81 mm Hg. The patient had a body mass index of 34 and on his abdomen pronounced striae distensae were visible. The electrocardiogram showed sinus rhythm with transmural ischaemia characterised by ST elevation in the anteroseptal leads and reciprocal ST depression at the inferior leads (Fig. 1). During coronary angiography, a complete occlusion was visible, at the ostium of the left anterior descending coronary artery (LAD) with collaterals from the right coronary artery (Fig. 2a). A percutaneous coronary intervention (PCI) was performed. A drug-eluting stent was placed in the LAD (Fig. 2b). Afterwards, cardiac ultrasound showed reasonable left ventricular function with an ejection fraction of 45%. The patient was discharged in a good clinical condition, with medication and lifestyle advice.

### Case 2

**Fig. 3:**
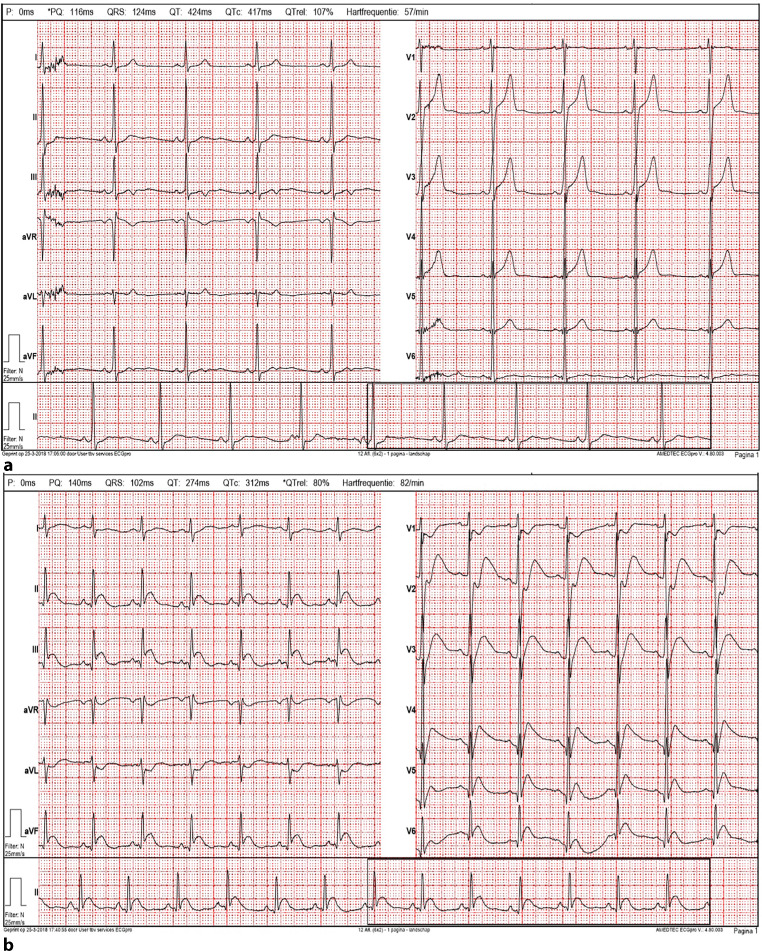
**a** Electrocardiogram: Non-specific intraventricular conduction delay with a QRS of 124 ms and symmetrical peaked T waves, suspicious for the hyperacute phase of ischaemia. **b** Electrocardiogram: convex ST elevations in the inferior and anterior wall

**Fig. 4:**
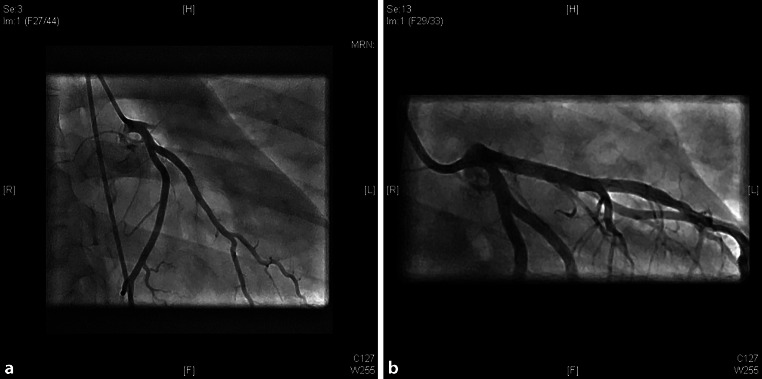
**a** Left anterior descending coronary artery, occlusion at the ostium. **b** Result immediately after percutaneous coronary intervention

A 27-year-old male was admitted to the hospital with complaints of pain in the chest radiating to both arms. In addition, the patient was nauseous and vomited. The complaints began a few minutes after he had performed motocross earlier the same day. The patient had no medical history. Cardiovascular disease at a young age did not occur in his family. He had never smoked, occasionally drank alcohol and did not use drugs. The patient had a blood pressure of 135/88 mm Hg. A troponin T test was negative; the C‑reactive protein level was low. His electrocardiogram showed a sinus rhythm with an intermediate heart axis and a prolonged QRS complex (124 ms) (Fig. 3a). Suddenly, ventricular fibrillation developed which required immediate resuscitation. After 10 min of resuscitation, a sinus rhythm was present with adequate cardiac output. The electrocardiogram showed precordial ST-segment elevation (Fig. 3b). Cardiac ultrasound showed a left ventricle ejection fraction of 56% with anterior wall movement disorders. Coronary angiography was performed and revealed a completely occluded LAD with no further signs of angiographic atherosclerosis (Fig. 4a). The operator refrained from further intravascular coronary imaging because of the life-threatening clinical condition and the fact that it would not have any effect on the treatment strategy in this young subject. The LAD blockade was treated with two drug-eluting stents (Fig. 4b). Hereafter, the patient was stabilised. He recovered in 4 days and was discharged from hospital.

## Point of view

It is important to consider an acute MI as a diagnosis in adolescents with chest pain. Acute MI can present with or without ST-segment elevations on the electrocardiogram: STEMI or non-STEMI [4, 5]. Prior to acute MI, a phase of unstable angina pectoris may occur. Unstable angina pectoris means chest pain at rest, de novo or with a crescendo pattern. Unstable angina pectoris, STEMI and non-STEMI are part of the acute coronary syndrome [4, 5]. It is important to realise that acute MI presents differently over time on an electrocardiogram. At first, there is a hyperacute phase with large symmetrical or peaked T waves. These are followed by convex ST-segment elevation, which represents a completely developed ischaemic phase. Finally, a chronically stabilised phase develops, in which negative T waves are replaced by positive T waves. A STEMI in adolescents may be the result of premature atherosclerosis or have an underlying non-atherosclerotic cause [1, 2, 6].

### Premature atherosclerosis

Development of coronary atherosclerosis at a young age is correlated with the presence of conventional cardiovascular risk factors. Examples are smoking, hypertension, dyslipidaemia, overweight, inactivity and stress [1]. Smoking is one of the most important risk factors. Frequent exposure to cigarette smoke stimulates the release of catecholamines, which cause damage to the endothelial cells. This can lead to vascular intima dysfunction already at a young age. This process involves alternating low-level cholesterol deposition and platelet aggregation (plaque formation). The lipid core within this plaque formation may rupture. This results in release of vasoactive factors leading to acute vascular occlusion. Genetic predisposition also increases the risk of premature atherosclerosis, such as in the case of a mutation in the factor V Leiden gene or genetic hypercholesterolaemia [7]. Treatment of acute MI due to premature atherosclerosis consists of protocolised revascularisation by means of primary PCI, followed by cardiovascular risk management on risk factors.

### Non-atherosclerotic causes

**Fig. 5:**
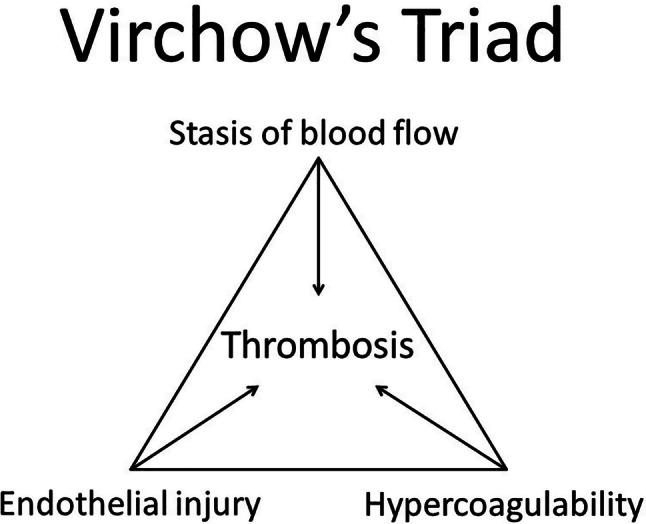
Virchow triad

In MI of non-atherosclerotic causes the mechanism of coronary occlusion differs [1, 2]. This may result from various underlying disorders that are accompanied by unconventional risk factors in some cases, such as pregnancy and direct contact sports. In Tab. 1, an overview of non-atherosclerotic causes is depicted. For example, the occlusion can be induced by coronary spasms, embolisation through coronary arteries due to endocarditis or secondary thrombus formation. Development of secondary thrombosis could be explained by the Virchow triad (Fig. 5; [8]). This theory describes that development of thrombosis that can be triggered by three factors: stasis of blood flow, endothelial damage and hypercoagulability or abnormal blood composition. For example, patients with the nephrotic syndrome have an increased risk of developing thrombosis because of an increased coagulation state. Coronary spasms can also cause an increased risk of secondary thrombosis because these spasms induce minor damage to the vascular endothelium and activate coagulation. Both cocaine and binge drinking can cause coronary spasms [1, 9, 10].

**Table 1:** Overview of mechanisms of a myocardial infarction (*MI*) with a non-atherosclerotic cause

Mechanism	Examples and explanation
Coronary artery spasms	Drug and excessive alcohol use *Use of cocaine and binge drinking are associated with pain in the chest, myocardial infarction, tachyarrhythmias and bradyarrhythmias. This is due to the acute rise in heart frequency and systemic blood pressure, with an increased oxygen requirement. Also, vasoconstriction of the coronary arteries occurs due to α*_*1*_*-adrenergic properties of cocaine and calcium-dependent vasoconstriction. Endothelial dysfunction predisposes to vasoconstriction and both cocaine and alcohol promote accelerated atherosclerosis *[9, 11]
Aberrant anatomy of the coronary arteries	Aberrant course between the aorta and pulmonary artery *An anomalous course of coronary arteries is correlated with narrowing of luminal diameters, promotion of atherosclerosis and may therefore cause ischaemia *[12]
Systemic inflammatory disease	Systemic lupus erythematosus, rheumatoid arthritis, Wegener granulomatosis *These are autoimmune diseases which can affect the myocardium, the cardiac valves, the conduction system, the pericardium and the coronary vascularisation. Regarding coronary vascularisation, the development of atherosclerosis may be accelerated due to chronic low-grade inflammation *[13]
Thrombosis	Endocarditis, coagulation disorders, nephrotic syndrome *In cases of endocarditis, myocardial infarction may occur due to septic vegetations on the aortic and mitral valves *[14]*. In patients with the nephrotic syndrome, clotting factors may be present, such as increased platelet aggregation, antithrombin III deficiency and changes in platelet function *[15]
Pregnancy	Change of haemodynamic state (increase in the amount of blood and coagulants), mucoid degeneration *Pregnancy increases blood volume by 140–150% and increases levels of factor VIII and von Willebrand factor. These are essential coagulation proteins. Simultaneously, levels of protein S are decreased (a vitamin-K-dependent anticoagulant). The change in blood composition decreases the risk of bleeding during childbirth, but increases the risk of developing thrombosis and subsequently the risk of a MI *[16]*. Mucoid degeneration of the intima in the coronary arteries due to pregnancy could lead to coronary ischaemia *[17]
Endothelial damage	Blunt chest trauma, spontaneous dissection of the coronary artery, potentially also in cases of deceleration trauma *Several mechanisms of coronary occlusion due to blunt chest trauma have been described: shear force at the coronary arteries may cause intimal tear and lead to intraluminal thrombosis. Also vascular ruptures, coronary artery embolism, fissuring of an atherosclerotic plaque with displacement of atherosclerotic plaques and vascular spasm may occur *[18]*. Potentially, similar mechanisms are involved in MI after deceleration trauma. A spontaneous dissection occurs due to separation of the coronary artery wall caused by an intimal tear and may result in medial dissection, haemorrhage and the formation of a false lumen. It is suggested that inflammation, hormonal factors and connective tissue diseases play a role in the development of a spontaneous dissection of the coronary artery *[19]

An occlusion of the coronary arteries could also be caused by damaged vascular endothelium after blunt trauma to the chest [20]. This may occur during direct physical contact and ball sports. The damage to the vascular endothelium may induce locally increased coagulation with formation of thrombosis. In addition, a blunt trauma to the chest can also cause coronary spasms or a rupture of an already existing plaque [20].

We argue that deceleration trauma may also result in acute MI. This might have been the cause in the second case. In this case, the patient repeatedly performed jumps with his motor cycle. Following these high jumps, the motocross bike lands with a relatively high impact. This might have induced tearing of the vascular endothelium and subsequently induced intraluminal thrombosis. Also, vascular ruptures, spasms, coronary artery embolism or displacement of an atherosclerotic plaque may occur in the case of deceleration trauma. Additionally, relative dehydration due to strenuous exercise could have increased the viscosity of the blood. This could also have contributed to acute red thrombus formation [21].

In young women, acute MI is rare because of the protective effect of oestrogens [22]. However, there are a few exceptions. First, the risk of developing acute MI is increased during and after pregnancy in the postpartum period (up to 356 days after delivery). This is due to several physiological changes. During pregnancy, there is an increase in blood volume of 140–150% and increased levels of factor VIII and von Willebrand factor. These are essential coagulation proteins. Simultaneously, levels of protein S are decreased (a vitamin-K-dependent anticoagulant). The change in blood composition decreases the risk of bleeding during childbirth, but increases the risk of developing thrombosis and subsequently the risk of acute MI [16]. Also, there is evidence that mucoid degeneration of the intima in the coronary arteries due to pregnancy could lead to coronary ischaemia [17]. Use of the contraceptive pill combined with smoking increases the risk of coronary ischaemia in women. Moreover, spontaneous dissection of the coronary arteries is more prevalent in women compared to men [19, 23].

In addition, it is important to realise that in some cases ST-segment elevations on an electrocardiogram may indicate another serious condition. This is called coronary mimicry, or the electrographic simulation of acute MI [24, 25]. The electrocardiogram may show ST elevations, negative T waves and QS complexes. Examples of diseases that could mimic STEMI are cerebrovascular accidents, a pneumothorax, pulmonary embolism, peri-myocarditis, pyelonephritis, pancreatitis, hyperkalaemia and metabolic acidosis.

In cases of acute MI with a non-atherosclerotic cause, treatment should be individualised with regard to the underlying mechanism. For example, coronary vascular spasm should be treated with non-specific vasodilators (e.g. nitrates) [26]. Adequate lifestyle advice should be provided, such as discouraging the use of cocaine and drinking large amounts of alcohol. In coronary mimicry, the underlying illness should be treated. It is important to realise that protocolised revascularisation by means of primary PCI may be fatal due to, for example, a delay in appropriate treatment or administration of medication with adverse effects. For example, the use of anticoagulants in cases of a haemorrhagic cerebrovascular accident might lead to life-threatening situations.

## Conclusion

It is important to consider acute MI as a potential diagnosis in adolescents with chest pain. Fortunately, in the aforementioned patients, the MI was recognised at an early stage and treated successfully. Both patients recovered without considerable residual damage. Early recognition of acute MI at a young age is mainly hampered by a paucity in understanding of the different potentially underlying pathophysiological mechanisms. Insight into the potential operational mechanisms of the coronary artery incident may have a major impact on the clinical course following admission. We would like to underline that a personalised clinical approach remains of utmost importance in each patient treated by protocolised medicine. This is particularly true when acute MI occurs at a young age, since the underlying cause more frequently differs from the conventional atherosclerotic process in this patient category.
